# Association between daily sitting time and osteoarthritis based on the National Health and Nutrition Examination Survey (NHANES) 2007 to 2018: A cross-sectional study

**DOI:** 10.1097/MD.0000000000047940

**Published:** 2026-03-06

**Authors:** Zhihao Chen, Wang Wei, Ruiying Li, Dazhi Wang, Zenan Tian, Yisen Feng, Dongjian Wang, Zhikun Jia, Jiajun Jiang, Xiaoyang Wang, Jianlong Ni, Qichun Song, Zhibin Shi

**Affiliations:** a Department of Sports Medicine and Pediatric Orthopaedics, The Second Affiliated Hospital of Xi’an Jiaotong University, Xi’an Jiaotong University, Xi’an, Shaanxi, PR China; b Third Department of Orthopaedics, Shaanxi Sengong Hospital, Xi’an, Shaanxi, PR China.

**Keywords:** cross-sectional study, NHANES, osteoarthritis, physical activity, risk factors, sedentary behavior, sitting time

## Abstract

Osteoarthritis (OA) affects over 500 million people globally, making it a significant public health concern. While various risk factors have been identified, the relationship between sedentary behavior and OA remains understudied. Data from the National Health and Nutrition Examination Survey 2007 to 2018 were analyzed. A total of 15,014 adults who met the inclusion criteria from 60,204 initial participants were included. Participants were categorized based on daily sitting time (≤9 hours vs >9 hours). Multiple logistic regression models were used to examine the association between sitting time and OA. Further analyses were stratified by participation in moderate recreational activities. The prevalence of OA was 8.9% (1329/15,014) in the study population. After adjusting for potential confounders, participants with >9 hours of daily sitting time had 36% increased risk of OA compared with those with ≤9 hours (95% confidence interval: 1.09–1.70, *P* < .05). This association was more pronounced among participants who did not engage in moderate recreational activities (odds ratio: 1.67, 95% confidence interval: 1.24–2.26, *P* < .001). Notably, the association between sitting >9 hours/day and OA was not significant among those who participated in moderate recreational activities. Subgroup analyses revealed that race and alcohol consumption might modify the relationship between sitting time and OA among physically inactive participants. Extended daily sitting time (>9 hours) is associated with an increased risk of OA, particularly among individuals who do not engage in moderate recreational activities. The finding that moderate recreational activities may modify the association between sitting >9 hours/day and OA warrants further investigation through prospective cohort studies.

## 1. Introduction

Osteoarthritis (OA), recognized as the most prevalent degenerative joint disease, impacts over 500 million individuals globally, constituting approximately 7% of the world’s population and thereby imposing a substantial public health burden.^[1,2]^ It is characterized by progressive degeneration of articular cartilage, subchondral bone changes, and osteophyte formation.^[3]^ Current therapeutic strategies primarily aim to mitigate the symptoms, and there are no satisfactory drugs or treatments that prevent or delay OA progression. Consequently, identifying potentially modifiable protective or risk factors is crucial for developing targeted interventions aimed at reducing the prevalence of OA within the population.

The link between sedentary behavior and chronic disease is well-established in the literature. Sedentary behaviors are defined as any waking activity performed while sitting, reclining, or lying down, with an energy expenditure of no more than 1.5 metabolic equivalents.^[4,5]^ It has been associated with an increased risk of several chronic diseases, including diabetes, cardiovascular disease, and obesity. Despite the scientific evidence and organizational efforts to promote physical activity, many people remain sedentary or do not engage in enough physical activity to achieve health benefits.^[6]^ A study by Henson et al highlighted the importance of reducing sedentary time in the management and prevention of type 2 diabetes mellitus.^[7]^ The authors emphasized the need to include reduced sitting time as a key component of diabetes management and prevention pathways. The impact of sedentary behavior on chronic disease has also been studied in the context of specific conditions. For example, Ramachandruni et al discussed the role of acute and chronic psychological stress in coronary heart disease, highlighting the potential link between stress, sedentary behavior, and cardiovascular health.^[8]^ In addition, Ayoub et al investigated the prevalence of medullary tophi in patients with chronic kidney disease, suggesting a possible link between uric acid levels, sedentary behavior, and progressive kidney disease.^[9]^

The relationship between sedentary behavior and OA remains a topic of interest in the field of musculoskeletal health. Existing literature provides insights into various aspects of OA, including loading patterns, inflammation, and senescence.^[10–12]^ Increased participation in sports or other physical activity has also been suggested to underlie the occurrence of high-level OA in previous studies.^[13]^ However, physical inactivity has become an important factor in the increased prevalence of OA over the past few decades, particularly in developed nations.^[14]^ It may contribute to the development of OA through a number of indirect factors, such as promoting obesity and metabolic validation, or telomere shortening.^[15,16]^ A study from a subcohort of the Osteoarthritis Initiative also supports the idea of alleviating physical functioning in people with OA by less sedentariness.^[17]^ Otherwise, there are few direct assessments of the potential impact of sitting time on the incidence of OA.

Therefore, the purpose of this study was to examine the association between sitting time and OA in US adults using national cross-sectional data. It also provides potential evidence for the evaluation of public health guidelines for the prevention of OA.

## 2. Materials and methods

### 2.1. Ethical approval

The research protocol was approved by the Ethics Review Board of the Centers for Disease Control and Prevention’s National Center for Health Statistics (NCHS). All study procedures strictly adhered to applicable institutional requirements and local regulations. As this study constitutes a secondary analysis of the existing National Health and Nutrition Examination Survey (NHANES) data – a publicly available dataset with preexisting ethical clearance – neither additional institutional review nor written informed consent from participants or their legal representatives was required. This study did not utilize any potentially identifiable personal information, as all analyzed data were fully deidentified in accordance with NHANES data dissemination policies.

### 2.2. Study population

**Figure 1.**
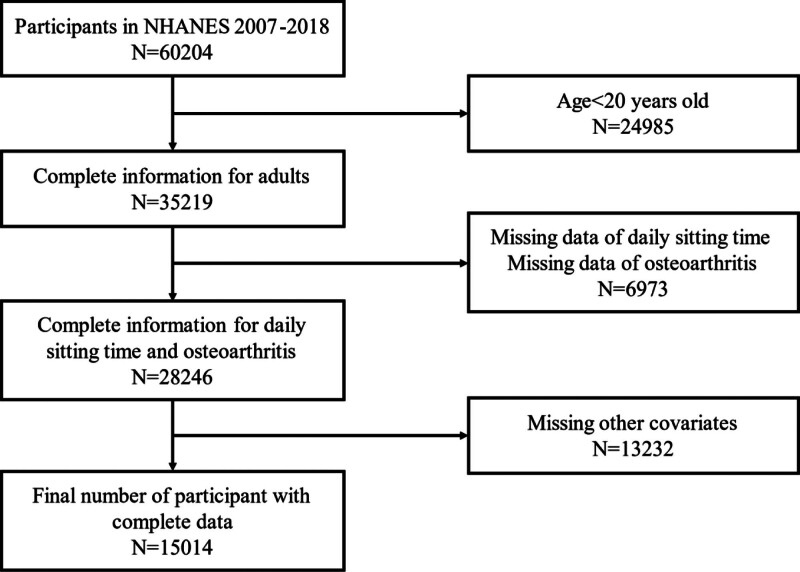
Sample selection flowchart from National Health and Nutrition Examination Survey (NHANES 2007–2018).

The NHANES is a large, complex, nationally representative health survey conducted by the NCHS in the United States. NHANES employs a stratified, multistage probability sampling design to select a nationally representative sample. The survey covers a wide range of data, including demographic characteristics, dietary intake, physical examinations, and laboratory tests. The data collected by NHANES pertain to chronic diseases and their risk factors, enabling the estimation of disease prevalence, the evaluation of public health policies and programs, and providing valuable data resources for related research. Six cycles of data in the NHANES database from 2007 to 2018 were utilized in this study. Participants who met the following inclusion criteria were included: aged ≥ 20 years, with complete data on daily sitting time and OA, and with data on other covariates (Fig. 1). Participants diagnosed with other arthritis types, such as rheumatoid arthritis, were excluded. Further information regarding ethical approvals and procedures for informed consent can be obtained from the NCHS. Comprehensive information on the design, methodology, and weighting of the NHANES has been previously published.^[18]^

### 2.3. Sitting time

Daily sitting time was the exposure variable in our study. The question is entitled “Minutes sedentary activity” and is measured by the question “How much time do you usually spend sitting on a typical day?” This variable is defined as “sitting at school, at home, getting to and from places, or with friends including time spent sitting at a desk, travelling in a car or bus, reading, playing cards, watching a video game, or sitting on a computer.” Participants were divided into 2 categories based on their daily sitting time: 9 hours or less per day and more than 9 hours. The highest category defined as >9 hours/day based on the restricted cubic spline (RCS) analysis inflection point (Fig. S1, Supplemental Digital Content, https://links.lww.com/MD/R502).

### 2.4. OA history

OA status was assessed and recorded in NHANES through the Medical Conditions Questionnaire. OA was identified by asking participants 2 key questions. First, they were asked “Has a doctor or other health professional ever told (you/she) that (you/she)… has arthritis?” If the answer was “yes,” a follow-up question was asked: “What type of arthritis was it?” Based on the answers to these questions, OA was distinguished from other types of arthritis, and participants were classified into the OA group (those with OA only), the other arthritis group (excluded from further analysis), and the no arthritis group. This self-reported physician-diagnosed OA is the most widely used case definition in epidemiological studies.^[19]^ The reliability of this approach is supported by a previous study showing an 81% agreement between self-reported OA and clinically confirmed OA.^[20]^ Thus, the data obtained in NHANES using this method provide a solid basis for the study of OA.

### 2.5. Covariates

Several additional covariates were collected and used for adjustment in this study to reduce potential bias. Based on the literature, the following covariates were included: age, sex, body mass index (BMI), race, education, family income ratio, smoking status, alcohol drinking status, diabetes, hypertension, cardiovascular disease, and moderate activity. According to the World Health Organization guidelines,^[21]^ BMI was divided into 4 grades: underweight (<18.5 kg/m^2^), normal weight (18.5–24.9 kg/m^2^), overweight (25–29.9 kg/m^2^), and obesity (≥30 kg/m^2^). As used by NHANES, we categorized the participants into the following 6 races and ethnicities: Mexican American, non-Hispanic Asian, non-Hispanic Black, non-Hispanic White, Other Hispanic, and Other (including multiracial). Educational level was divided into 3 levels (high school or less, some college, and college graduate or higher). As used by US government departments to report NHANES dietary and health data,^[22]^ we categorized family income into the following 3 levels based on the family poverty income ratio: low income (≤1.3), medium income (>1.3 to 3.5), and high income (>3.5).^[23]^ Smoking status was categorized by reported cigarettes smoked^[24]^: participants who reported smoking <100 cigarettes in their lifetime were defined as a “never smoker”; participants who reported smoking ≥100 cigarettes in their lifetime and were currently smoking at the time of the questionnaire either “every day” or “some days” were defined as a “current smoker”; and participants who reported smoking ≥100 cigarettes in their lifetime and had quit smoking at the time of the questionnaire were defined as a “former smoker.” As the 2015 to 2020 Dietary Guidelines for Americans defined alcohol intake ≥1 drink/day in women and ≥2 drinks/day in men as MODERATE,^[25]^ we categorized alcohol consumption as below moderate and above moderate groups.

### 2.6. Statistical analysis

The statistical analyses were conducted in accordance with the guidelines established by the Centers for Disease Control and Prevention. Participants with missing covariate data were excluded from the analysis. Appropriate sample weights were utilized in the recruitment of participants and the execution of statistical analyses. The baseline characteristics of the enrolled population were documented, and the subjects were categorized into groups with and without OA. Categorical variables were expressed as numbers and the corresponding weighted proportions, and their association was examined using the survey-weight chi-square test. In order to analyze the association between daily sitting time and OA, a weighted logistic regression analysis was conducted, with the following models: first, a crude model; second, model 1 that was adjusted for age, race, education level, and family income-to-poverty ratio; and third, model 2 that was additionally adjusted for smoking status, alcohol consumption status, diabetes, hypertension, cardiovascular disease, and moderate recreational activity. The strength of the association was estimated by odds ratios (ORs) and the associated 95% confidence intervals (CIs). To examine the potential nonlinear dose-response relationship between sedentary time and OA, we utilized RCSs with 3 knots. The reference level for the RCS analysis was set at 4 hours/day.

To further explore the relationship between daily sitting time and OA, we stratified the analyses according to “moderate recreational activity” or not. A subgroup analysis was performed to identify variables that affect the association between sitting time and OA in the no moderate recreational activity subgroup. In order to test for interaction effects between covariates and daily sitting time and to assess the robustness of the results, a further stratified logistic regression analysis and likelihood ratio test were performed. The results of this analysis are presented as *P* for interaction. The analyses were conducted utilizing the R statistical software, version 4.1.3 (http://www.R-project.org; The R Foundation, Vienna, Austria). The survey package and “dplyr” package of R were used to conduct the weighted analysis. A significance level of *P* < .05 (2-tailed) was designated as the criterion for statistical significance.

## 3. Results

### 3.1. Population characteristics

From 2007 to 2018, a total of 60,204 individuals were initially recruited in the survey. A total of 15,014 participants aged ≥20 years with complete information were included in our study for further analysis (Fig. 1). The prevalence of OA was 8.9% (1329/15,014). The demographics and characteristics of participants in this study are shown in Table 1. We found that OA was more common in older people (*P* < .001) and women (*P* < .001), and the proportion of females with OA was higher than the proportion of males (58.2% vs 41.8%). The proportion of obese patients in the OA group was significantly higher than in the non-OA group (50.1% vs 43.2%), and the proportion of non-Hispanic White also higher significantly (55.2% vs 43.3%). Smoking, alcohol consumption, and participation in moderate activities are strongly associated with the prevalence of OA (*P* < .001). The prevalence of common chronic diseases such as diabetes mellitus, hypertension, and cardiovascular diseases was significantly higher in the OA group compared with the non-OA group (*P* < .001).

**Table 1:** Characteristics of the study population from NHANES 2007 to 2018, weighted.

Characteristics	OA		*P*-value
	No	Yes	
N	13,685 (91.1%)	1329 (8.9%)	
Sitting time/d (h)			.556
≤9	11,510 (84.1%)	1109 (83.4%)	
>9	2175 (15.9%)	220 (16.6%)	
Sex			<.001
Male	7559 (55.2%)	556 (41.8%)	
Female	6126 (44.8%)	773 (58.2%)	
Age (yr)			<.001
20–29	3524 (25.8%)	24 (1.8%)	
30–39	3078 (22.5%)	99 (7.4%)	
40–49	2631 (19.2%)	170 (12.8%)	
50–59	1991 (14.5%)	304 (22.9%)	
60–69	1491 (10.9%)	364 (27.4%)	
70–79	681 (5.0%)	274 (20.6%)	
≥80	289 (2.1%)	94 (7.1%)	
BMI (kg/m^2^)			<.001
<18.5	213 (1.6%)	16 (1.2%)	
18.5–24.9	4071 (29.7%)	286 (21.5%)	
25.0–29.9	4670 (34.1%)	361 (27.2%)	
≥30	4731 (43.6%)	666 (50.1%)	
Race			<.001
Mexican American	2239 (16.4%)	121 (9.1%)	
Non-Hispanic White	5922 (43.3%)	733 (55.2%)	
Non-Hispanic Black	2707 (19.8%)	269 (20.2%)	
Other Hispanic	1396 (10.2%)	110 (8.3%)	
Other race	1421 (10.4%)	96 (7.2%)	
Educational level			.496
Below high school	2644 (19.3%)	255 (19.2%)	
High school or equivalent	2970 (21.7%)	271 (20.4%)	
College graduate or above	8071 (59.0%)	803 (60.4%)	
Family income-to-poverty ratio			.594
≤1.3	3817 (27.9%)	380 (28.6%)	
1.3–3.5	5147 (37.6%)	481 (36.2%)	
≥3.5	4721 (34.5%)	468 (35.2%)	
Hypertension			<.001
No	10,449 (76.4%)	566 (42.6%)	
Yes	3236 (23.6%)	763 (57.4%)	
Smoking status			<.001
Current	3323 (24.3%)	307 (23.1%)	
Former	2864 (20.9%)	484 (36.4%)	
Never	7498 (54.8%)	538 (40.5%)	
Diabetes			<.001
No	12,726 (93.0%)	1056 (79.5%)	
Yes	959 (7.0%)	273 (20.5%)	
Alcohol consumption status			<.001
Moderate – no	7498 (54.8%)	551 (41.5%)	
Moderate – yes	6187 (45.2%)	778 (58.5%)	
Cardiovascular disease			<.001
No	13,412 (98.0%)	1221 (91.9%)	
Yes	273 (2.0%)	108 (8.1%)	
Moderate activity			<.001
No	7376 (53.9%)	796 (59.9%)	
Yes	6309 (46.1%)	533 (40.1%)	

BMI = body mass index, NHANES = National Health and Nutrition Examination Survey, OA = osteoarthritis.

### 3.2. Associations of daily sitting time and OA

Table 2 presents the association between daily sitting time and OA risk across multiple regression models. In the demographically adjusted model 1 (controlling for age, race, education level, and income-to-poverty ratio), participants with >9 hours of daily sitting time demonstrated a 36% increased risk of OA compared with those sitting ≤9 hours (OR: 1.36, 95% CI: 1.09–1.70, *P* < .01). This significant association persisted in the fully adjusted model 2, which additionally controlled for smoking status, alcohol consumption, diabetes, hypertension, cardiovascular disease, and moderate recreational activity (OR: 1.26, 95% CI: 1.01–1.58, *P* < .05). The results of sensitivity analysis were shown in model 3 (Table S1, Supplemental Digital Content, https://links.lww.com/MD/R502).

**Table 2:** Logistic regression analysis of sedentary for OA in participants ≥20 years old in NHANES (2007–2018), weighted.

Sedentary	Crude model	Model 1	Model 2
	OR (95% CI), *P*	OR (95% CI), *P*	OR (95% CI), *P*
≤9 h	Reference	Reference	Reference
>9 h	1.12 (0.91–1.37), .28	1.36 (1.09–1.70), **.007**	1.26 (1.01–1.58), **.04**

Bold values indicate statistical significance (*P* < .05).

Crude model: adjusted for none.

Model 1: adjusted for age, race, education level, and family income-to-poverty ratio.

Model 2: adjusted for age, race, education level, family income ratio, smoking status, alcohol consumption status, diabetes, hypertension, cardiovascular disease, and moderate recreational activity. *P* < .05 presents significant difference.

CI = confidence interval, NHANES = National Health and Nutrition Examination Survey, OR = odds ratio.

The RCS analysis visualized a nonlinear positive association between daily sitting time and OA risk after adjusting for confounders (Fig. S1, Supplemental Digital Content, https://links.lww.com/MD/R502). The risk of OA remained relatively stable at lower levels of sedentary behavior but showed a significant upward trend with sitting >9 hours/day. Notably, the OR became statistically significant (lower 95% CI > 1.0) when daily sitting time exceeded 8.4 hours, validating the rationale for identifying individuals sitting >9 hours/day as a high-risk population.

Stratification by physical activity revealed a critical interaction effect (Table 3). Among physically inactive participants, sitting >9 hours/day was associated with substantially elevated OA risk in all models, reaching a maximum increase of 67% in the demographic-adjusted model (OR: 1.67, 95% CI: 1.24–2.26, *P* < .001). Notably, the association between sitting >9 hours/day and OA was not observed among participants who engaged in moderate recreational activities.

**Table 3:** Univariate and multivariate analyses by activity-stratified logistic regression model, weighted.

Moderate activity	Sedentary	
	≤9 h	>9 h
	OR (95% CI), *P*	OR (95% CI), *P*
No		
Crude model	Reference	1.56 (1.19–2.03), **.001**
Model 1	Reference	1.67 (1.24–2.26), **<.001**
Model 2	Reference	1.49 (1.10–2.03), **.01**
Yes		
Crude model	Reference	0.74 (0.53–1.02), 0.06
Model 1	Reference	1.01 (0.72–1.44), 0.93
Model 2	Reference	0.97 (0.68–1.38), 0.8

Bold values indicate statistical significance (*P* < .05).

Crude model: adjusted for none.

Model 1: adjusted for age, race, education level, and family income-to-poverty ratio.

Model 2: adjusted for age, race, education level, family income-to-poverty ratio, smoking status, alcohol consumption status, diabetes, hypertension, cardiovascular disease. *P* < .05 presents significant difference.

CI = confidence interval, OR = odds ratio.

### 3.3. Subgroup analysis

To elucidate potential effect modifiers in the sedentary behavior-OA relationship, we conducted stratified logistic regression analyses and formal interaction tests among physically inactive participants (Table 4). Among multiple demographic and clinical variables examined, only race (*P* < .05 for interaction) and alcohol consumption (*P* < .05 for interaction) demonstrated statistically significant effect modification. Specifically, the association between sitting >9 hours/day and OA was particularly pronounced among non-Hispanic Whites (OR: 1.71, 95% CI: 1.20–2.44) compared with other racial/ethnic groups. Similarly, individuals reporting moderate alcohol consumption exhibited a stronger sitting-OA association (OR: 2.44, 95% CI: 1.54–3.85) than those with lower consumption levels. No significant interactions were detected for other covariates, including age, gender, BMI, education level, income, hypertension, smoking status, diabetes, or cardiovascular disease, suggesting the robustness of the primary association across these subpopulations. These findings indicate that racial background and alcohol consumption patterns may influence the pathophysiological mechanisms through which sedentary behavior contributes to OA risk in physically inactive individuals.

**Table 4:** Stratified logistic regression analysis to identify variables that modify the correlation between sitting time and OA in moderate activity subgroup, weighted.

Characteristics	Sedentary		*P* for interaction
	≤9 h	>9 h	
	OR (95% CI)	OR (95% CI)	
Sex			
Male	Reference	1.957 (1.18–3.246)	.228
Female	Reference	1.319 (0.905–1.921)	
Age (yr)			.569
20–29	Reference	1.181 (0.147–9.486)	
30–39	Reference	2.59 (1.195–5.616)	
40–49	Reference	1.748 (0.71–4.299)	
50–59	Reference	1.831 (1.044–3.21)	
60–69	Reference	1.582 (0.831–3.013)	
70–79	Reference	1.131 (0.538–2.377)	
≥80	Reference	0.688 (0.227–2.086)	
BMI (kg/m^2^)			.135
<18.5	Reference	0.513 (0.001–212.641)*	
18.5–24.9	Reference	1.119 (0.566–2.213)	
25.0–29.9	Reference	1.194 (0.656–2.174)	
≥30	Reference	1.869 (1.229–2.842)	
Race			<.05
Mexican American	Reference	1.506 (0.536–4.237)	
Non-Hispanic White	Reference	1.713 (1.201–2.443)	
Non-Hispanic Black	Reference	0.85 (0.497–1.455)	
Other Hispanic	Reference	0.724 (0.202–2.595)	
Other race	Reference	0.287 (0.068–1.218)	
Educational level			.592
Below high school	Reference	1.741 (0.866–3.498)	
High school or equivalent	Reference	1.17 (0.555–2.466)	
College graduate or above	Reference	1.58 (1.077–2.318)	
Family income-to-poverty ratio			.503
≤1.3	Reference	1.031 (0.574–1.851)	
1.3–3.5	Reference	1.642 (1.037–2.601)	
≥3.5	Reference	1.561 (0.936–2.604)	
Hypertension			.895
No	Reference	1.454 (0.902–2.344)	
Yes	Reference	1.528 (1.029–2.27)	
Smoking status			.263
Current	Reference	2.479 (1.444–4.256)	
Former	Reference	1.335 (0.813–2.192)	
Never	Reference	1.263 (0.73–2.186)	
Diabetes			.259
No	Reference	1.386 (0.973–1.976)	
Yes	Reference	2.142 (1.089–4.214)	
Alcohol consumption status			<.05
Moderate – no	Reference	1.056 (0.705–1.58)	
Moderate – yes	Reference	2.436 (1.542–3.848)	
Cardiovascular disease			.817
No	Reference	1.510 (1.098–2.075)	
Yes	Reference	0.975 (0.351–2.709)	

BMI = body mass index, CI = confidence interval, OA = osteoarthritis, OR = odds ratio.

* The wide confidence interval observed in the underweight group (BMI < 18.5 kg/m^2^) is due to the small sample size and limited number of OA events in this subgroup. This estimate should be interpreted with caution.

## 4. Discussion

To our knowledge, this study represents one of the pioneering investigations to systematically examine the association between sedentary behavior and OA utilizing large-scale cross-sectional data from the NHANES dataset. While the relationship between physical activity patterns and OA has been explored previously, our research makes a distinct contribution by establishing a semiquantitative dose-response relationship between sedentary behavior and OA outcomes. After adjusting for key demographic variables (model 1), individuals sitting more than 9 hours daily exhibited up to a 36% increased risk of OA compared with those sitting ≤9 hours (OR: 1.36, 95% CI: 1.09–1.70, *P* = .007), representing the maximum potential risk elevation observed in our models. Notably, after further adjustment for smoking status, alcohol consumption, and chronic diseases (model 2), this association remained statistically significant despite slight attenuation (OR: 1.26, 95% CI: 1.01–1.58, *P* = .04). This relationship demonstrated a distinctive pattern of effect modification by physical activity levels. Among physically inactive participants, the magnitude of the association strengthened considerably, with sitting >9 hours/day linked to a 67% increased OA risk (OR: 1.67, 95% CI: 1.24–2.26, *P* < .001). Our stratified analyses further demonstrated that this increased risk was primarily observed among individuals who did not engage in moderate recreational activities, while the significant association between sitting >9 hours/day and OA was attenuated and not statistically significant in those who regularly participated in moderate activity. These findings align with the established concept of hormesis in exercise physiology, where appropriate mechanical loading provides protective conditioning that increases tissue resilience to stress.^[26]^ Further subgroup analyses revealed additional effect modifiers, with race and alcohol consumption significantly influencing the sedentary behavior-OA relationship specifically among physically inactive individuals.

Growing evidence suggests that sedentary behavior is a significant risk factor for various chronic diseases. Previous studies have linked excessive sedentary time to conditions such as kidney stones,^[27]^ abdominal aortic calcification,^[28]^ and depression in chronic kidney disease patients.^[29]^ These findings underscore the importance of investigating sedentary behavior as a novel lifestyle factor in chronic disease etiology. In the context of OA, the rising prevalence has been attributed to increased participation in sports and athletic activities that often result in intra-articular stress or trauma.^[13,30]^ However, this hypothesis remains controversial. An alternative explanation for the increasing OA prevalence over the past few decades, particularly in developed countries, points to physical inactivity as a more likely culprit.^[14]^ This perspective raises important questions about the potential causal relationship between physical inactivity and heightened OA risk, as well as the underlying mechanisms driving this association.

Our current study, utilizing nationally representative NHANES data, provides critical insights into the relationship between sedentary behavior and OA. While prior research using NHANES data established a sedentary behavior classification threshold of >7.5 hours/day, which was statistically derived from the lowest quintile (Q1) cutoff versus higher quintiles (Q2–Q5) of average total sedentary time reported by participants,^[22]^ our study employed a risk threshold relevant to the OA population. Through RCS analysis, we observed that the risk of OA becomes statistically significant starting from 8.4 hours/day. Based on this data-driven finding, we defined >9 hours/day as the cutoff. This threshold not only captures the population with significantly elevated risk but also provides a clear, actionable metric for clinical screening and public health interventions, distinguishing it from lower thresholds used for metabolic conditions. This observation represents an important step forward, as it identifies a more precise, clinically relevant cut-point at which OA prevalence rises significantly. Healthcare providers may use this actionable threshold to stratify high-risk patients and implement targeted interventions. Moreover, this specific parameter can guide future mechanistic studies exploring the pathophysiological links between sitting >9 hours/day and joint degeneration.

Our study addresses a notable gap in the current OA literature by quantifying the sedentary behavior-OA association in a large, diverse sample. The observed dose-response relationship, with daily sitting over 9 hours conferring markedly higher OA risk compared with lower thresholds, corroborates and extends previous findings. These include a Mendelian randomization analysis identifying television viewing as a risk factor for knee and spinal OA,^[31]^ and Osteoarthritis Initiative data suggesting functional improvements in less sedentary OA patients.^[17]^ Importantly, our stratified analyses revealed that moderate recreational activity acts as a potential effect modifier, attenuating the potential risk associated with sitting >9 hours/day. This finding offers valuable insight for designing preventive strategies in susceptible populations. However, it is important to note some methodological limitations when interpreting these results. For instance, in the study by He et al,^[32]^ inadequate adjustment for potential confounders in the model construction may have influenced the reliability of their conclusions. Careful consideration of key covariates is essential to minimize residual confounding and enhance the validity of observed associations in observational studies.

Few studies have investigated the association between sedentary behavior and OA. Lee et al found that adults with knee OA spent approximately two-thirds of their daily time in sedentary behavior, which was significantly associated with poorer physical function.^[17]^ Similarly, Kaur et al reported a high prevalence of knee OA among women with sedentary lifestyles, highlighting the role of inactivity as a determinant of this condition.^[33]^ A systematic review by Zhao et al indicated that each additional hour of sedentary time was associated with increased risks of chronic diseases, including OA, underscoring the importance of reducing sedentary behavior to mitigate health risks.^[34]^ The present study’s results suggest that sitting for more than 9 hours per day significantly increases the risk of developing OA by 26% to 36%.

Several potential mechanisms may explain the observed association between sedentary behavior and OA. First, appropriate mechanical loading is essential for maintaining the structural integrity of articular cartilage and surrounding tissues.^[35]^ This relationship follows a “use-or-lose” pattern,^[36]^ where prolonged sedentary behavior leads to a reduction in these necessary mechanical stimuli, which may compromise joint lubrication and cartilage homeostasis.^[37,38]^ Second, sedentary behavior is closely linked to muscle atrophy and reduced muscle strength, which are critical for joint stability.^[39,40]^ The weakening of periarticular muscles can increase joint loading and accelerate wear. Finally, sedentary behavior contributes to obesity and chronic low-grade inflammation,^[41–43]^ both of which are established risk factors that may accelerate OA progression through metabolic pathways.^[44]^ However, as this is an observational study, further research is needed to elucidate these specific causal pathways.

The current study, which employed data from the NHANES, has several noteworthy strengths. The NHANES dataset, being a nationally representative sample of the US population, offers a solid and diverse basis for examining the associations between sedentary behavior and OA across a broad spectrum of demographic groups. The complex sampling design of NHANES helps to minimize selection bias, ensuring that the study sample accurately represents the target population. The substantial sample size, exceeding 15,000 participants, enhances the study’s statistical power, facilitating the identification of significant associations while minimizing the risk of type II errors. Furthermore, the study adopted a semiquantitative approach to investigate the relationship between sedentary time and OA, providing a more granular understanding of the potential dose-response relationship between these variables. The analysis also took into account crucial covariates, such as age, gender, and levels of recreational activity, which could potentially confound the association between sedentary behavior and OA, thereby enhancing the reliability of the findings. Future research should focus on elucidating the specific pathways through which sedentary behavior contributes to OA development and progression, as well as identifying effective interventions to mitigate these risks. Longitudinal studies with objective measures of sedentary behavior, physical activity, and joint health can provide valuable insights into the causal relationships and potential dose-response effects. Additionally, investigating the role of lifestyle modifications, such as breaking up sedentary with light activity or incorporating targeted exercise programs, in preventing and managing OA in sedentary individuals can inform evidence-based guidelines and public health strategies.

Certain limitations of the study should be taken into consideration when interpreting the results. The cross-sectional design hinders the establishment of a causal relationship between sedentary behavior and OA, as it is not feasible to ascertain whether sedentary behavior directly contributes to the development of OA or if individuals with OA are more inclined to engage in sedentary behavior due to their condition. Moreover, the assessment of sedentary behavior and recreational activity levels relied on self-reported data, which may be prone to recall bias and social desirability bias. Third, due to the design of the NHANES questionnaire, we were unable to differentiate the specific affected joint sites (e.g., knee, hip, or hand OA). While the association between sedentary behavior and OA is most biologically plausible for weight-bearing joints, the lack of site-specific data prevents stratified analysis. However, given that knee and hip OA represent the most prevalent forms of OA in the general population, our findings likely reflect, to a significant extent, the relationship between sedentarism and weight-bearing joint health. Future studies utilizing radiographic data or site-specific diagnoses are warranted to validate these findings. Additionally, while the NHANES dataset is extensive, it may not capture all pertinent variables that could influence the relationship between sedentary behavior and OA, such as occupational physical activity, dietary habits, and genetic predisposition. Finally, the study’s focus on US population may limit the generalizability of the findings to other countries or cultures with different lifestyle patterns and risk factors for OA. Despite these limitations, the present study offers valuable insights into the association between sedentary behavior and OA, emphasizing the association between sitting >9 hours/day and OA, which appears to be modified by moderate recreational activity.

## 5. Conclusion

In summary, findings from a nationally representative sample of US adults suggest that sedentary time >9 hours per day is associated with a significantly increased risk of developing OA and that this risk increases when combined with no moderate recreational activity. However, participation in moderate recreational activities may modify the association between daily sedentary time and OA that we found in the group without moderate recreational activities. Further prospective cohort studies will be conducted in the future to test this association.

## Acknowledgments

We thank all the researchers and officers from the National Health and Nutrition Examination Survey (NHANES).

## Author contributions

**Conceptualization:** Zhibin Shi.

**Methodology:** Zhikun Jia.

**Data curation:** Ruiying Li.

**Formal analysis:** Zhihao Chen, Dazhi Wang, Zenan Tian, Dongjian Wang.

**Investigation:** Yisen Feng.

**Resources:** Jiajun Jiang.

**Software:** Xiaoyang Wang.

**Validation:** Jianlong Ni.

**Visualization:** Qichun Song.

**Project administration:** Zhibin Shi.

**Writing – original draft:** Zhihao Chen.

**Writing – review & editing:** Wang Wei.

## Supplementary Material



## References

[1] Duan WL Zhang LN Bohara R. Adhesive hydrogels in osteoarthritis: from design to application. Mil Med Res. 2023;10:4. 36710340 10.1186/s40779-022-00439-3 PMC9885614

[2] Katz JN Arant KR Loeser RF. Diagnosis and treatment of hip and knee osteoarthritis: a review. JAMA. 2021;325:568–78. 33560326 10.1001/jama.2020.22171 PMC8225295

[3] Jiang W Jin Y Zhang S. PGE2 activates EP4 in subchondral bone osteoclasts to regulate osteoarthritis. Bone Res. 2022;10:27. 35260562 10.1038/s41413-022-00201-4 PMC8904489

[4] Tremblay MS Aubert S Barnes JD. Sedentary behavior research network (SBRN) – terminology consensus project process and outcome. Int J Behav Nutr Phys Act. 2017;14:75. 28599680 10.1186/s12966-017-0525-8 PMC5466781

[5] Chau JY Grunseit AC Chey T. Daily sitting time and all-cause mortality: a meta-analysis. PLoS One. 2013;8:e80000. 24236168 10.1371/journal.pone.0080000 PMC3827429

[6] Myers J Atwood JE Froelicher V. Active lifestyle and diabetes. Circulation. 2003;107:2392–4. 12756188 10.1161/01.CIR.0000067882.00596.FC

[7] Henson J Dunstan DW Davies MJ Yates T. Sedentary behaviour as a new behavioural target in the prevention and treatment of type 2 diabetes. Diabetes Metab Res Rev. 2016;32:213–20. 26813615 10.1002/dmrr.2759

[8] Ramachandruni S Handberg E Sheps DS. Acute and chronic psychological stress in coronary disease. Curr Opin Cardiol. 2004;19:494–9. 15316459 10.1097/01.hco.0000132321.24004.25

[9] Ayoub I Almaani S Brodsky S. Revisiting medullary tophi: a link between uric acid and progressive chronic kidney disease? Clin Nephrol. 2016;85:109–13. 26709523 10.5414/CN108663

[10] Thorp LE Sumner DR Wimmer MA Block JA. Relationship between pain and medial knee joint loading in mild radiographic knee osteoarthritis. Arthritis Rheum. 2007;57:1254–60. 17907211 10.1002/art.22991

[11] Collins KH Paul HA Reimer RA Seerattan RA Hart DA Herzog W. Relationship between inflammation, the gut microbiota, and metabolic osteoarthritis development: studies in a rat model. Osteoarthritis Cartilage. 2015;23:1989–98. 26521745 10.1016/j.joca.2015.03.014

[12] Liu Y Zhang Z Li T Xu H Zhang H. Senescence in osteoarthritis: from mechanism to potential treatment. Arthritis Res Ther. 2022;24:174. 35869508 10.1186/s13075-022-02859-x PMC9306208

[13] Palmieri-Smith RM Cameron KL DiStefano LJ. The role of athletic trainers in preventing and managing posttraumatic osteoarthritis in physically active populations: a consensus statement of the athletic trainers’ osteoarthritis consortium. J Athl Train. 2017;52:610–23. 28653866 10.4085/1062-6050-52.2.04 PMC5488853

[14] Hallal PC Andersen LB Bull FC Guthold R Haskell W Ekelund U. Global physical activity levels: surveillance progress, pitfalls, and prospects. Lancet. 2012;380:247–57. 22818937 10.1016/S0140-6736(12)60646-1

[15] Arsenis NC You T Ogawa EF Tinsley GM Zuo L. Physical activity and telomere length: impact of aging and potential mechanisms of action. Oncotarget. 2017;8:45008–19. 28410238 10.18632/oncotarget.16726 PMC5546536

[16] Berenbaum F Wallace IJ Lieberman DE Felson DT. Modern-day environmental factors in the pathogenesis of osteoarthritis. Nat Rev Rheumatol. 2018;14:674–81. 30209413 10.1038/s41584-018-0073-x

[17] Lee J Chang RW Ehrlich-Jones L. Sedentary behavior and physical function: objective evidence from the Osteoarthritis Initiative. Arthritis Care Res. 2015;67:366–73. 10.1002/acr.22432 PMC4336845 25155652

[18] Botman S Moriarity CL. Design and estimation for the national health interview survey, 1995–2004. https://stacks.cdc.gov/view/cdc/6518. Accessed January 11, 2025.

[19] Xu Y Wu Q. Trends and disparities in osteoarthritis prevalence among US adults, 2005–2018. Sci Rep. 2021;11:21845. 34750468 10.1038/s41598-021-01339-7 PMC8576014

[20] Li Y Zhu J Fan J. Associations of urinary levels of phenols and parabens with osteoarthritis among US adults in NHANES 2005–2014. Ecotoxicol Environ Saf. 2020;192:110293. 32045785 10.1016/j.ecoenv.2020.110293

[21] Obesity and overweight. https://www.who.int/news-room/fact-sheets/detail/obesity-and-overweight. Accessed January 11, 2025.

[22] Almohamad M Krall Kaye E Mofleh D Spartano NL. The association of sedentary behaviour and physical activity with periodontal disease in NHANES 2011–2012. J Clin Periodontol. 2022;49:758–67. 35634657 10.1111/jcpe.13669

[23] A quick guide to SNAP eligibility and benefits | Center on Budget and Policy Priorities. October 1, 2015. https://www.cbpp.org/research/food-assistance/a-quick-guide-to-snap-eligibility-and-benefits. Accessed January 11, 2025.

[24] Tomar SL Asma S. Smoking-attributable periodontitis in the United States: findings from NHANES III. J Periodontol. 2000;71:743–51. 10.1902/jop.2000.71.5.743 29537517

[25] 2015-2020 dietary guidelines | odphp.health.gov. https://odphp.health.gov/our-work/nutrition-physical-activity/dietary-guidelines/previous-dietary-guidelines/2015. Accessed February 26, 2026.

[26] Ji LL Kang C Zhang Y. Exercise-induced hormesis and skeletal muscle health. Free Radic Biol Med. 2016;98:113–22. 26916558 10.1016/j.freeradbiomed.2016.02.025

[27] Li Y Di X Liu M Wei J Li T Liao B. Association between daily sitting time and kidney stones based on the national health and nutrition examination survey (NHANES) 2007–2016: a cross-sectional study. Int J Surg. 2024;110:4624–32. 38768465 10.1097/JS9.0000000000001560 PMC11325893

[28] Sheng C Huang W Wang W Lin G Liao M Yang P. The association of moderate-to-vigorous physical activity and sedentary behaviour with abdominal aortic calcification. J Transl Med. 2023;21:705. 37814346 10.1186/s12967-023-04566-w PMC10563258

[29] Liu L Yan Y Qiu J. Association between sedentary behavior and depression in US adults with chronic kidney disease: NHANES 2007–2018. BMC Psychiatry. 2023;23:148. 36894924 10.1186/s12888-023-04622-1 PMC9996893

[30] Pinto D Song J Lee J. Association between sedentary time and quality of life from the Osteoarthritis Initiative: who might benefit most from treatment? Arch Phys Med Rehabil. 2017;98:2485–90. 28645770 10.1016/j.apmr.2017.06.004 PMC13102485

[31] Wang Y Zhang Y Zhao C Cai W Wang Z Zhao W. Physical activity, sedentary behavior, and osteoarthritis: a two-sample mendelian randomization analysis. Iran J Public Health. 2023;52:2099–108. 37899916 10.18502/ijph.v52i10.13848 PMC10612556

[32] He J Zhang C Yang L. Association between sedentary behavior, physical activity, and osteoarthritis: results from NHANES 2007–2020 and Mendelian randomization analysis. Front Public Health. 2025;12:1454185. 39872105 10.3389/fpubh.2024.1454185 PMC11771141

[33] Kaur R Ghosh A Singh A. Prevalence of knee osteoarthritis and its determinants in 30–60 years old women of gurdaspur, punjab. Int J Med Sci Public Health. 2018;7:825.

[34] Zhao R Bu W Chen Y Chen X. The dose-response associations of sedentary time with chronic diseases and the risk for all-cause mortality affected by different health status: a systematic review and meta-analysis. J Nutr Health Aging. 2020;24:63–70. 31886810 10.1007/s12603-019-1298-3 PMC12879226

[35] Griffin TM Guilak F. The role of mechanical loading in the onset and progression of osteoarthritis. Exerc Sport Sci Rev. 2005;33:195–200. 16239837 10.1097/00003677-200510000-00008

[36] Urquhart DM Tobing JFL Hanna FS. What is the effect of physical activity on the knee joint? A systematic review. Med Sci Sports Exerc. 2011;43:432–42. 20631641 10.1249/MSS.0b013e3181ef5bf8

[37] Matthews CE George SM Moore SC. Amount of time spent in sedentary behaviors and cause-specific mortality in US adults. Am J Clin Nutr. 2012;95:437–45. 22218159 10.3945/ajcn.111.019620 PMC3260070

[38] Shirazi R Shirazi-Adl A Hurtig M. Role of cartilage collagen fibrils networks in knee joint biomechanics under compression. J Biomech. 2008;41:3340–8. 19022449 10.1016/j.jbiomech.2008.09.033

[39] Upadhyay SS Moulton A Srikrishnamurthy K. An analysis of the late effects of traumatic posterior dislocation of the hip without fractures. J Bone Joint Surg Br. 1983;65-B:150–2. 10.1302/0301-620X.65B2.6826619 6826619

[40] Magalhães E Fukuda TY Sacramento SN Forgas A Cohen M Abdalla RJ. A comparison of hip strength between sedentary females with and without patellofemoral pain syndrome. J Orthop Sports Phys Ther. 2010;40:641–7. 20508327 10.2519/jospt.2010.3120

[41] van den Bosch MHJ Blom AB van der Kraan PM. Inflammation in osteoarthritis: our view on its presence and involvement in disease development over the years. Osteoarthritis Cartilage. 2024;32:355–64. 38142733 10.1016/j.joca.2023.12.005

[42] Martínez-González MA Alfredo Martínez J Hu FB Gibney MJ Kearney J. Physical inactivity, sedentary lifestyle and obesity in the European Union. Int J Obes. 1999;23:1192–201. 10.1038/sj.ijo.0801049 10578210

[43] Henson J Yates T Edwardson CL. Sedentary time and markers of chronic low-grade inflammation in a high risk population. PLoS One. 2013;8:e78350. 24205208 10.1371/journal.pone.0078350 PMC3812126

[44] Gupta UC Gupta SC Gupta SS. Clinical overview of arthritis with a focus on management options and preventive lifestyle measures for its control. Curr Nutr Food Sci. 2022;18:476–86.

