# Supplementary material for: Association between daily sitting time and osteoarthritis based on the National Health and Nutrition Examination Survey (NHANES) 2007 to 2018: A cross-sectional study
Source: Medicine (Baltimore). 2026 Mar 6;105(10):e47940. doi: 10.1097/MD.0000000000047940 (PMC12975173; doi:10.1097/MD.0000000000047940)

***Supplementary Material***

***Supplementary Table 1*** Logistic regression analysis of Sedentary for OA in participants ≥ 20 years old in NHANES (2007–2018), weighted (Model 3)

| **Sedentary** | **Model 3** |
| --- | --- |
|  | OR(95%CI), P |
| **<=9h** | Reference |
| **>9h** | 1.22(0.97-1.53),  0.08 |

Model 3: adjusted for age, race, education level, family income ratio, **BMI**, smoking status, alcohol consumption status, diabetes, hypertension, cardiovascular disease, and moderate recreational activity. p < 0.05 presents significant difference. OR, Odds ratio; CI, Confidence interval; BMI, Body mass index

**Supplementary Figure 1.** Restricted cubic spline analysis of the association between daily sitting time and osteoarthritis. The model was adjusted for age, sex, race, BMI, education, PIR, smoking, alcohol, diabetes, hypertension, cardiovascular disease, and moderate activity. The solid blue line represents the multivariate-adjusted odds ratio (OR), and the shaded area represents the 95% confidence interval. The dashed vertical line indicates the 9-hour threshold. The analysis shows that the lower bound of the 95% CI exceeds 1.0 at approximately 8.4 hours/day.


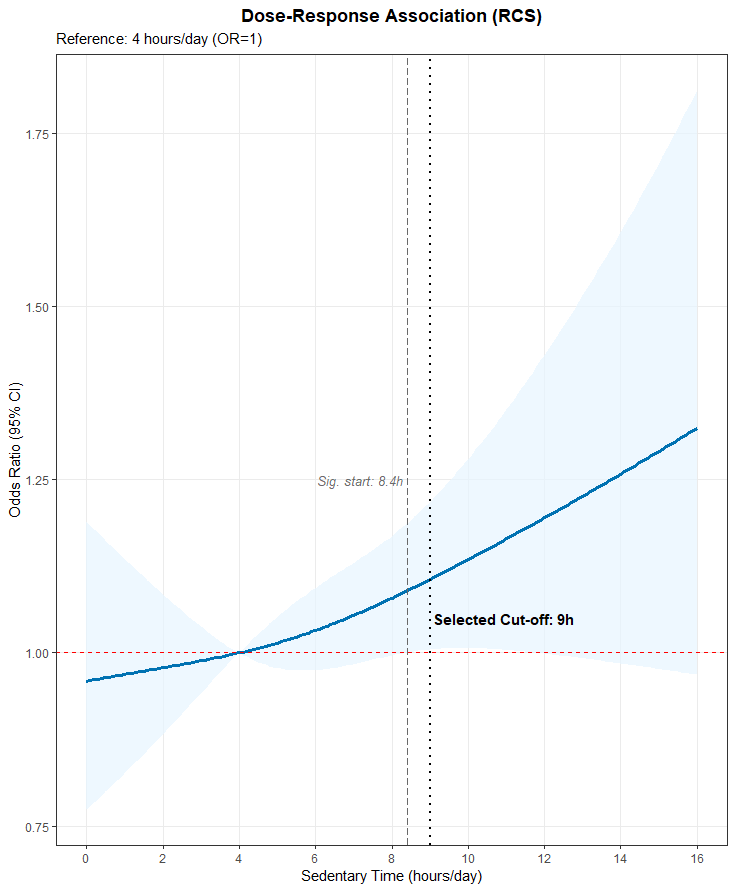

Supplement: Supplementary file 1 [file medi-105-e47940-s001.docx]
